# Neural Substrates of Spontaneous Musical Performance: An fMRI Study of Jazz Improvisation

**DOI:** 10.1371/journal.pone.0001679

**Published:** 2008-02-27

**Authors:** Charles J. Limb, Allen R. Braun

**Affiliations:** 1 Language Section, Voice, Speech and Language Branch, National Institute on Deafness and Other Communication Disorders, National Institutes of Health, Bethesda, Maryland, United States of America; 2 Department of Otolaryngology-Head and Neck Surgery and Peabody Conservatory of Music, Johns Hopkins University, Baltimore, Maryland, United States of America; University of Southern California, United States of America

## Abstract

To investigate the neural substrates that underlie spontaneous musical performance, we examined improvisation in professional jazz pianists using functional MRI. By employing two paradigms that differed widely in musical complexity, we found that improvisation (compared to production of over-learned musical sequences) was consistently characterized by a dissociated pattern of activity in the prefrontal cortex: extensive deactivation of dorsolateral prefrontal and lateral orbital regions with focal activation of the medial prefrontal (frontal polar) cortex. Such a pattern may reflect a combination of psychological processes required for spontaneous improvisation, in which internally motivated, stimulus-independent behaviors unfold in the absence of central processes that typically mediate self-monitoring and conscious volitional control of ongoing performance. Changes in prefrontal activity during improvisation were accompanied by widespread activation of neocortical sensorimotor areas (that mediate the organization and execution of musical performance) as well as deactivation of limbic structures (that regulate motivation and emotional tone). This distributed neural pattern may provide a cognitive context that enables the emergence of spontaneous creative activity.

## Introduction

A significant number of recent studies have used functional neuroimaging methods to investigate the perception of musical stimuli by the human brain [1]–[10]. The broad appeal of these studies is likely to be related to the universal nature of music throughout history and across cultures, as well as the intrinsic relationship between music and language. Fewer studies, however, have examined the central mechanisms that give rise to music performance [11], [12] while, to our knowledge, only one other study [13] has examined the neural substrates that give rise to the spontaneous production of novel musical material, a process that extends well beyond the technical or physical requirements of musical production *per se*. Spontaneous musical performance, whether through singing or playing an instrument, can be defined as the immediate, on-line improvisation of novel melodic, harmonic, and rhythmic musical elements within a relevant musical context. Most importantly, the study of spontaneous musical improvisation may provide insights into the neural correlates of the creative process.

Creativity is a quintessential feature of human behavior, but the neural substrates that give rise to it remain largely unidentified. Spontaneous artistic creativity is often considered one of the most mysterious forms of creative behavior, frequently described as occurring in an altered state of mind beyond conscious awareness or control [14]–[16] while its neurophysiological basis remains obscure. Here we use functional neuroimaging methods to examine musical improvisation as a prototypical form of spontaneous creative behavior, with the assumption that the process is neither mysterious nor obscure, but is instead predicated on novel combinations of ordinary mental processes. It has been suggested that the prefrontal cortex is a region of critical importance that enables the creative process (which includes self-reflection and sensory processing as integral components) [14]. We hypothesized that spontaneous musical improvisation would be associated with discrete changes in prefrontal activity that provide a biological substrate for actions that are characterized by creative self-expression in the absence of conscious self-monitoring. Furthermore, we hypothesized that alterations in prefrontal cortical activity would be associated with top-down changes in other systems, particularly sensorimotor areas needed to organize the on-line execution of musical ideas and behaviors, as well as limbic structures needed to regulate memory and emotional tone.

In this study, we used functional MRI to study improvisation, which is the hallmark of jazz music [17]. During a jazz performance, musicians utilize a composition's underlying chord structure and melody as the contextual framework and basis upon which a novel solo is extemporaneously improvised. Hence, no two jazz improvisations are identical. The process of improvisation is involved in many aspects of human behavior beyond those of a musical nature, including adaptation to changing environments, problem solving and perhaps most importantly, the use of natural language, all of which are unscripted behaviors that capitalize on the generative capacity of the brain.

**Figure 1:**
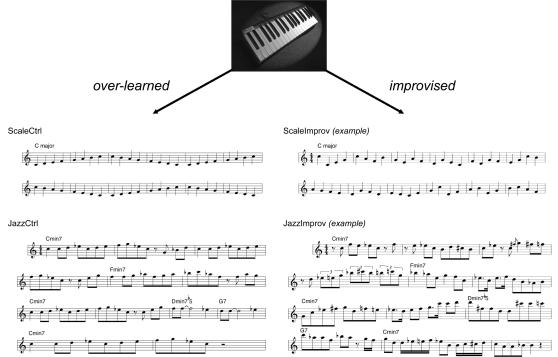
Low complexity (Scale) and high complexity (Jazz) experimental paradigms used to study spontaneous musical creativity. In the upper portion of the figure, the non-ferromagnetic MIDI piano keyboard that was used during functional MRI scanning is shown. This keyboard had thirty five full-size piano keys which triggered high-quality piano sound samples generated outside of the scanner, which were immediately routed back to the musicians using audiophile quality electrostatic earphone speakers. During scanning, subjects were randomly cued to play either the over-learned control condition or to improvise spontaneously. For Scale's control condition, subjects repeatedly played a one octave ascending and descending C major scale in quarter notes for the duration of the block (ScaleCtrl, upper left). For Scale's improvisation condition, subjects improvised in quarter notes only, selecting all notes from within one octave and from the C major scale notes alone (example shown under ScaleImprov, upper right). For Jazz's control condition, subjects played a novel melody that was memorized prior to scanning (JazzCtrl, lower left). For Jazz's improvisation condition, subjects improvised using the composition's underlying chord structure as the basis for spontaneous creative output (example shown under JazzImprov, lower right). Note that for JazzCtrl and JazzImprov, eighth notes are typically performed with a “swing” feel that is not accurately represented using standard musical notation, in both the control and improvisation conditions. Audio samples of the four musical excerpts shown here are provided in Supporting Information.

Since musical improvisation is an extraordinarily complex human behavior, we felt that it should be examined using paradigms that, while amenable to experimental constraint, are of high ecological validity (as argued by Burgess and colleagues; see [18], [19]. We therefore designed such a paradigm—that of professional jazz pianists improvising on a piano keyboard during image acquisition, alone and with the musical accompaniment of a jazz quartet—using tasks of similar ecological validity to control for the perceptual and motor features of performance. Six highly skilled professional jazz musicians underwent functional MR brain scans (3 Tesla) during which they played a non-ferromagnetic piano keyboard specially designed for use in an fMRI setting (Fig. 1, upper). Because musical improvisation incorporates a broad range of melodic, harmonic, and rhythmic invention that is intrinsically difficult to control (while retaining musical integrity), we designed two paradigms, one that was relatively low (which we have termed Scale) and one that was high (which we have termed Jazz) in musical complexity. Both utilized musical control tasks designed to engage the same sensorimotor circuits but to generate pre-determined, over-learned output.

In Scale's control condition (referred to hereafter as ScaleCtrl), subjects repeatedly played a one-octave C major scale in quarter notes. During the corresponding improvisation condition (referred to as ScaleImprov), subjects improvised a melody, but were restricted to the use of C major scale quarter notes within the same octave. In the Jazz paradigm, we aimed to reproduce the high degree of musical richness of a jazz performance. Subjects were asked to memorize an original jazz composition (Fig. 1, lower left) several days prior to the study. During the control condition (referred to hereafter as JazzCtrl), subjects played the composition with the auditory accompaniment of a pre-recorded jazz quartet. During the corresponding improvisation condition (referred to as JazzImprov), subjects were given freedom to improvise, using the chord structure of the composition and the same auditory accompaniment as the basis for improvisation.

All notes were recorded using MIDI (Musical Instrument Digital Interface) technology and measures derived from these recordings—total number, rate and range of musical notes and finger/hand movements—were statistically compared off-line. Thus, for each paradigm, motor activity and lower level auditory features in both conditions could be matched, with the only difference being whether the musical output was improvised or over-learned (see Audio S1, Audio S2, Audio S3, and Audio S4 in Supporting Information). Comparing these paradigms should make it possible to study not simply the content of creativity (in this case, the specific musical output during improvisation), but more importantly, the neural correlates of the cognitive state in which spontaneous creativity unfolds.

## Results

### MIDI Data Analysis

The statistical analysis of piano MIDI performance data by paired T-tests revealed no significant difference between total number or weighted distribution of notes played during improvisation or control conditions for either Scale or Jazz paradigms (Table 1). During the Scale paradigm, there was no difference between subjects in absolute range of notes played (highest or lowest note) for ScaleCtrl or ScaleImprov and no statistical difference between weighted distributions of notes. During the Jazz paradigm, there was a statistically insignificant difference in absolute range of notes for both minimum (mean of 2 notes lower) and maximum (mean of 6 notes higher) between JazzCtrl and JazzImprov, because subjects were free to improvise, but no difference in weighted distribution of notes during these conditions.

**Table 1:** MIDI piano data obtained during control and improvisation conditions for Scale and Jazz paradigms.

	Scale			Jazz		
	Control	Improv	*p*	Control	Improv	*p*
**Number of notes** [mean (s.d.)]	348.67 (1.03)	349.17 (1.47)	0.076	755.33 (20.76)	787 (184.7)	0.66
**Weighted distribution of notes** [mean (s.d.)]	23.45 (0.01)	23.53 (0.20)	0.37	23.13 (0.12)	24.55 (1.76)	0.11

Data in Table 1 are shown in mean ±standard deviation, with two-tailed paired t-test results.

### Functional MRI Data Analysis

Functional imaging data were analyzed using SPM99 through standard contrasts (and inclusive masking where appropriate), conjunctions between paradigms, and comparison of hemodynamic response functions (see Experimental Procedures for further details). In order to be deemed significant, clusters of activation associated with improvisation were required to demonstrate both greater activity levels vs. resting baseline as well as greater activity levels vs. control conditions; clusters of deactivation were required to show both lower activity levels vs. resting baseline as well as lower activity levels vs. control conditions. This additional masking allowed us to distinguish true experimental activations from relative activations caused by deactivation during the control condition.

**Figure 2:**
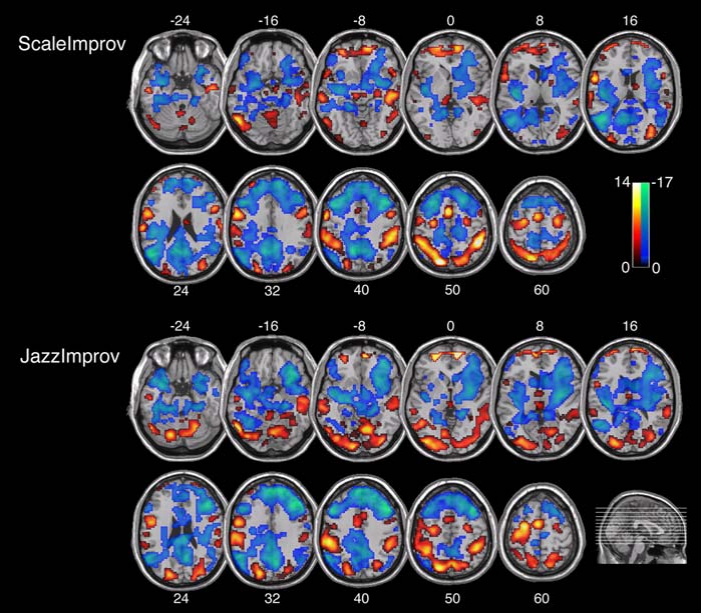
Axial slice renderings of mean activations (red/yellow scale bar) and deactivations (blue/green scale bar) associated with improvisation during Scale and Jazz paradigms. In both paradigms, spontaneous improvisation was associated with widespread deactivation in prefrontal cortex throughout DLPFC and LOFC, combined with focal activation in MPFC. In addition, increases in sensorimotor activity and decreases in limbic activity were seen in both paradigms. Activations were identified through inclusive masking of the contrast for [Improv–Control] with the contrast for [Improv–Rest], and deactivations were identified through inclusive masking of the contrast for [Control–Improv] with the contrast for [Rest–Improv] for both Scale and Jazz paradigms. The scale bar shows t-score values and the sagittal section shows an anatomical representation of slice location; both scale bar and sagittal slice insets apply equally to Scale and Jazz data. Labels refer to axial slice z-plane in Talairach space.

**Figure 3:**
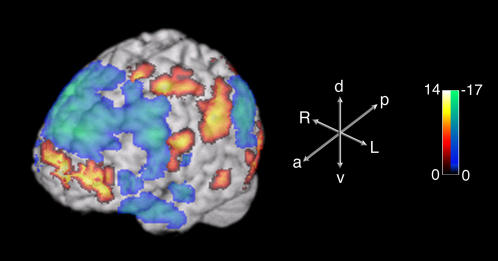
Three-dimensional surface projection of activations and deactivations associated with improvisation during the Jazz paradigm. Medial prefrontal cortex activation, dorsolateral prefrontal cortex deactivation, and sensorimotor activation can be seen. The scale bar shows the range of t-scores; the axes demonstrate anatomic orientation. Abbreviations: a, anterior; p, posterior; d, dorsal; v, ventral; R, right; L, left.

Both paradigms yielded strikingly similar results (Fig. 2, Table 2). Spontaneous improvisation was in each case associated with a highly congruous pattern of activations and deactivations in prefrontal cortex, sensorimotor and limbic regions of the brain (Figs. 2 and 3). In addition, the majority of these regions showed functionally reciprocal patterns of activity. That is, activations during improvisation were matched by deactivations during the control tasks, and vice versa, when each condition was compared to the resting baseline. The major findings are described below:

**Table 2:** Local maxima and minima of brain activations and deactivations within the prefrontal cortex during improvisation.

Region	BA	Left Hemisphere				Right Hemisphere			
		t-score	*x*	*y*	*z*	t-score	*x*	*y*	*z*
**Activations**									
*Medial Prefrontal*									
Polar MPF-ventral	10	-	-	-	-	15.97	12	57	−6
Polar MPF-middle	10	11.26	−27	53	−2	11.26	7	61	3
Polar MPF-dorsal	10	15.68	−27	63	15	14.04	3	63	12
**Deactivations**									
*Medial Prefrontal*									
Dorsal MPFC	8,9	−16.23	−12	48	36	−18.15	12	51	33
*Dorsolateral Prefrontal*									
Medial DLPFC	46	−7.441	−30	41	34	−14.71	51	30	27
Lateral DLPFC	9	−22.05	−42	21	39	−20.79	39	24	39
Superor DLPFC	8	−15.67	−36	18	51	−12.81	41	17	53
*Lateral Orbitofrontal*									
Ventral LOFC	47,11	-	-	-	-	−11.42	33	21	−24
Mid LOFC	11	−14.81	−45	42	−15	−13.51	33	39	−15

All coordinates are described according to the Montreal Neurological Institute system, and were obtained using a conjunction analysis of data from ScaleImprov and JazzImprov. Activations (positive t-scores) and deactivations (negative t-scores) are shown. Abbreviations: BA, Brodmann Area; MPFC, medial prefrontal cortex; DLPFC, dorsolateral prefrontal cortex; LOFC, lateral orbitofrontal cortex

1. Within the prefrontal cortex, a dissociated pattern of activity was seen during improvisation. This was characterized by widespread deactivation that included almost all of the lateral prefrontal cortices, extending from lateral orbitofrontal cortex (LOFC) to the superior portions of the dorsolateral prefrontal cortex (DLPFC), as well as dorsal portions of the medial prefrontal cortex (MPFC). However, this broad pattern of deactivation was also accompanied by focal activation of the frontal polar portion of the MPFC (Fig. 2, Fig. 3, Table 2).
2. Broad increases in sensorimotor activity were associated with improvisation. In neocortical sensory areas, activations were seen in anterior portions of superior and middle temporal gyri (STG and MTG), including anterior portions of the superior temporal sulcus (STS), inferior temporal, fusiform and lateral occipital gyri, as well as inferior and superior parietal lobules and the intervening intraparietal sulci. In neocortical premotor and motor areas, selective activation during improvisation was seen in both ventral and dorsal lateral premotor areas, supplementary motor area and portions of the primary motor cortex. The anterior cingulate cortex, cingulate motor area, right lateral cerebellar hemisphere, and vermis were activated as well (Fig. 2, 3, and Table 3).
3. Widespread attenuation of activity in limbic and paralimbic regions was seen during improvisation. Selective deactivations were in this case detected in the amygdala, entorhinal cortex, temporal pole, posterior cingulate cortex, parahippocampal gyri, hippocampus and hypothalamus (Fig. 2, 3 and Table 4).

**Table 3:** Local maxima of brain activations within sensorimotor, cingulate, and cerebellar regions during improvisation.

Region	BA	Left Hemisphere				Right Hemisphere			
		t-score	*x*	*y*	*z*	t-score	*x*	*y*	*z*
**Sensorimotor**									
*Premotor/Motor*									
Frontal operculum-p. triangularis	45	6.51	−51	33	3	-	-	-	-
Frontal operculum-p. opercularis	44	11.42	−52	8	17	-	-	-	-
Dorsal frontal operculum	44/6	16.54	−54	0	30	7.14	51	6	27
Dorsal Lateral PMC	4/6	11.34	−30	−15	64	9.18	30	−6	63
SMA proper	6	16.93	−3	0	63	10.39	3	−4	68
Dorsal MI	4	14.58	−27	−15	54	10.04	27	−9	51
*Temporal*									
STG	22	-	-	-	-	6.14	63	−33	9
Ant MTG-STS	21	11.19	−63	−27	−9	10.72	57	−21	−9
Ant MTG-ITG	20/21	10.39	−51	−15	−24	6.41	45	−15	−18
Fusiform-ITG	37	15.74	−48	−66	−21	-	-	-	-
*Parietal*									
SMG	40	11.34	−53	−41	41	12.44	48	−41	41
IPS	40/7	16.05	−42	−45	45	17.46	45	−42	51
SPL	7	20.62	−18	−75	51	14.09	21	−77	55
*Occipital*									
Inf OG	18	7.01	−36	−90	−5				
Mid OG	18/19	7.87	−27	−94	14	11.18	36	−75	18
Sup OG	19	10	−21	−94	29	7.638	35	−83	25
**Cingulate**									
ACC D	32/24	10.71	−5	8	49	-	-	-	-
**Cerebellum**									
Dentate	-	-	-	-	-	7.96	21	−63	−30
Post Hemisphere	-	-	-	-	-	7.94	3	−78	−39
Vermis	-	-	-	-	-	6.22	6	−67	−17

All coordinates are described according to the Montreal Neurological Institute system, and were obtained through a conjunction analysis of data from ScaleImprov and JazzImprov. **Abbreviations:** p. triangularis, pars triangularis; p. opercularis, pars opercularis; PMC, premotor cortex; SMA, supplementary motor area; STG, superior temporal gyrus; MTG, middle temporal gyrus; STS, superior temporal sulcus; ITG, inferior temporal gyrus; SMG, supramarginal gyrus; IPS, intraparietal sulcus; SPL, superior parietal lobule; OG, occipital gyrus; ACC, anterior cingulate commissure

**Table 4:** Local minima of brain deactivations within limbic, basal ganglia, insula, and heteromodal sensory regions during improvisation.

Region	BA	Left Hemisphere				Right Hemisphere			
		t-score	*x*	*y*	*z*	t-score	*x*	*y*	*z*
**Limbic/Paralimbic**									
Hypothalamus	-	−8.51	−11	−6	−9	−11.12	9	−6	−6
Amygdala	-	−14.64	−24	0	−18	−7.205	28	−1	−16
HPC/PHPC-ventral	-	-	-	-	-	−12.49	27	−24	−18
HPC/PHPC-dorsal	-	−9.71	−24	−36	−3	−10.06	15	−42	3
PHPC gyrus	35,36	−13.08	−36	−27	−21	−11.34	24	−27	−24
Posterior cingulate	23,31	−13.92	−3	−51	24	−18.14	3	−57	30
Temporal polar	38,20	−14.27	−30	3	−24	−12.99	33	0	−39
**Basal ganglia**									
Ventral striatum	-	−15.41	−30	−12	−9	−13.12	27	6	−9
Caudate	-	−7.03	−12	15	11	−10.37	9	15	3
Putamen	-	−6.61	−29	−3	6	−10.76	27	−15	6
**Insula**									
Ant insula/pyriform cortex	-	−6.61	−33	15	−11	−10.86	33	15	12
Mid Insula	-	−6.38	−33	4	13	−11.11	33	0	12
Post Insula	-	−12.85	−33	−24	9	−6.02	42	−11	7
**Heteromodal sensory**									
Posterior STS	21	−17.77	−51	−57	18				
Angular gyrus	39	−17.12	−45	−69	30	−9.98	51	−54	24

All coordinates are described according to the Montreal Neurological Institute system, and were obtained through a conjunction analysis of data from ScaleImprov and JazzImprov. **Abbreviations:** HPC, hippocampal cortex; PHPC, parahippocampal cortex; Ant, anterior; Post, posterior; STS, superior temporal sulcus

As highly trained professional right-handed jazz pianists constitute a relatively select study population, the present study was limited to six musicians. To address the issue of a small sample size, we also utilized a multi-subject conjunction analysis to examine functional imaging data obtained from the piano improvisation experiments [20]. This method increases the statistical rigor of a fixed effects analysis for sample sizes that do not permit meaningful random effects analysis (as is the case here), and addresses the possibility that a single subject (or minority of subjects) is “driving” the fixed effects analysis. Results of this conjunction analysis, which are particularly stringent for focal activations (because voxels must be commonly activated in all six subjects to survive the conjunction), were consistent with those of the fixed effects analysis, with widespread deactivation in DLPFC, increased sensorimotor activity, and decreased limbic activity seen in all six subjects for both low and high complexity paradigms, and focal activation in MPF in five of six subjects (Jazz paradigm) and four of six subjects (Scale paradigm) (see Supporting Information, Fig. S1).

## Discussion

Our results strongly implicate a distinctive pattern of changes in prefrontal cortical activity that underlies the process of spontaneous musical composition. Our data indicate that spontaneous improvisation, independent of the degree of musical complexity, is characterized by widespread deactivation of lateral portions of the prefrontal cortex together with focal activation of medial prefrontal cortex. This unique pattern may offer insights into cognitive dissociations that may be intrinsic to the creative process: the innovative, internally motivated production of novel material (at once rule based and highly structured) that can apparently occur outside of conscious awareness and beyond volitional control.

In jazz music, improvisation is considered to be a highly individual expression of an artist's own musical viewpoint [17]. The association of MPFC activity with the production of autobiographical narrative [21] is germane in this context, and as such, one could argue that improvisation is a way of expressing one's own musical voice or story [17], [22]. In this sense, activity of the MPFC during improvisation is also consistent with an emerging view that the region plays a role in the neural instantiation of self, organizing internally motivated, self-generated, and stimulus-independent behaviors [23]–[25]. The portion of the MPFC that was selectively activated during improvisation, the frontal polar cortex (Brodmann Area 10), remains poorly understood but appears to serve a broad-based integrative function, combining multiple cognitive operations in the pursuit of higher behavioral goals [26], in particular adopting and utilizing rule sets that guide ongoing behavior [27]–[29] and maintaining an overriding set of intentions while executing a series of diverse behavioral subroutines [30]. All of these functions are necessarily required during the task of improvisation.

In comparison, the lateral prefrontal regions (LOFC and DLPFC), which were deactivated during improvisation, are thought to provide a cognitive framework within which goal-directed behaviors are consciously monitored, evaluated and corrected. The LOFC may be involved in assessing whether such behaviors conform to social demands, exerting inhibitory control over inappropriate or maladaptive performance [31]. The DLPFC, on the other hand, is thought to be responsible for planning, stepwise implementation and on-line adjustment of behavioral sequences that require retention of preceding steps in working memory [32]. The DLPFC is active, for example, during effortful problem-solving, conscious self-monitoring and focused attention [33], [34].

In light of these distinct roles, we believe that the dissociation of activity in MPFC and LOFC/DLPFC observed here during improvisation is highly meaningful. If increased activity in the MPFC serves as an index of internally motivated behavior, concomitant decreases in the LOF and DLPFC suggest that self-generated behaviors (such as improvisation) occur here in the absence of the context typically provided by the lateral prefrontal regions. Whereas activation of the lateral regions appears to support self-monitoring and focused attention, deactivation may be associated with defocused, free-floating attention that permits spontaneous unplanned associations, and sudden insights or realizations [35]. The idea that spontaneous composition relies to some degree on intuition, the “ability to arrive at a solution without reasoning” [36], may be consistent with the dissociated pattern of prefrontal activity we observed. That is, creative intuition may operate when an attenuated DLPFC no longer regulates the contents of consciousness, allowing unfiltered, unconscious, or random thoughts and sensations to emerge. Therefore, rather than operating in accordance with conscious strategies and expectations, musical improvisation may be associated with behaviors that conform to rules implemented by the MPFC outside of conscious awareness [27]. Indeed, in other domains it has been shown that focused attention and conscious self-monitoring can inhibit spontaneity and impair performance [37], [38]. In short, musical creativity vis-à-vis improvisation may be a result of the combination of intentional, internally generated self-expression (MPFC-mediated) with the suspension of self-monitoring and related processes (LOFC- and DLPFC-mediated) that typically regulate conscious control of goal-directed, predictable, or planned actions.

While the results of some previous studies [39] suggest that decreased activity in the DLPFC may indicate a reduction in working memory demands, we feel that this is unlikely here (indeed, it could be argued that improvisation places a greater demand upon working memory mechanisms than the routinized musical performance characterizing our control conditions). Since we minimized working memory demands in both paradigms–utilizing over-learned control tasks as well as experimental conditions in which subjects were relatively free to improvise–we suggest that attenuation of activity in the DLPFC in the present instance more likely reflects a reduction in the prefrontal mechanisms outlined above.

It has also been suggested that deactivation of the lateral prefrontal regions represents the primary physiologic change responsible for altered states of consciousness such as hypnosis, meditation or even daydreaming [15]. This is interesting in that jazz improvisation, as well as many other types of creative activity, have been proposed to take place in an analogously altered state of mind [16]. Moreover, a comparable dissociated pattern of activity in prefrontal regions has been reported to occur during REM sleep [40], a provocative finding when one considers that dreaming is exemplified by a sense of defocused attention, an abundance of unplanned, irrational associations and apparent loss of volitional control, features that may be associated with creative activity during wakefulness as well [41].

Since improvisation was also accompanied by changes in sensorimotor and limbic systems, it is tempting to speculate that these changes might be causally related, triggered in a top-down fashion by changes initiated in the prefrontal cortex. Increased activity in some of the sensory areas involved might be explained by their role in processing complex stimuli in the auditory modality. For example, the anterior temporal regions (anterior STG, MTG, and intervening STS) that were selectively activated during improvisation appear to play an integral role in processing complex features of highly structured acoustic stimuli, including music [42]. However, we observed similar increases in other sensory areas as well. While some of these increases may simply reflect task-related processing in other modalities during improvisation, co-activation of multiple sensory areas also suggests the intriguing possibility that musical spontaneity is associated with a generalized intensification of activity in all sensory modalities. This possibility is supported by our findings of widespread activation of neocortical motor systems even though the analysis of MIDI data revealed no significant differences in number or distribution of piano notes played during improvised or control conditions. Therefore, rather than reflecting an increase in motor activity *per se*, these activations may be associated with encoding and implementation of novel motor programs that characterize spontaneous improvisation.

Previous studies of music perception have reported both increases and decreases in limbic activity. Because of the presumed relationship between musical creativity and emotion, involvement of the limbic system was anticipated here. The deactivation of the amygdala and hippocampus we observed may be attributable to the positive emotional valence associated with improvisation, consistent with studies that have reported these limbic structures to be less active during perception of music that is consonant [4] or elicits intense pleasure [2]. However, we also observed more extensive deactivation of limbic structures in the hypothalamus, ventral striatum, temporal pole, and orbital cortex. The role played by these structures during improvisation will require further study.

In an intriguing neuroimaging study of musical improvisation in classically trained pianists, Bengtsson et al. [13] found activations in the right dorsolateral prefrontal cortex, as well as premotor and auditory areas during improvisation. Our study differs from this one in several important ways. First, the study by Bengtsson et al. utilized contrasts that were designed to remove deactivations. In comparison, we had the explicit goal of identifying relevant deactivations that might support the notion of a hypofrontal state associated with creative activity. Hence, the masking strategies employed by our studies were fundamentally different, and would be expected to lead to divergent results. Second, our subjects were jazz pianists (rather than classical pianists). This difference is relevant in that jazz, much more so than classical music, is intrinsically characterized by improvisation. As a result, we believe that our findings reflect neural mechanisms behind improvisation in a perhaps more natural context, and certainly in musicians who have finely developed improvisational skills. Lastly, Bengtsson and coworkers utilized conditions in which musical improvisations were generated and then subsequently reproduced by memory. These conditions address an interesting facet of improvisation—the interaction between spontaneous musical performance and memory. We sought to eliminate the secondary impact of episodic memory encoding on improvisation by using either an over-learned or completely improvised condition (without a reproduction task in either condition).

Because our experiments were performed in highly trained musicians, it remains to be clarified whether or not our findings have characterized a higher qualitative level of musical output (as opposed to that which might be produced by less skilled performers). However, the similar findings seen for both Scale and Jazz paradigms, despite the musical simplicity of the former, strongly suggest that our findings are attributable to neural mechanisms that underlie spontaneity more broadly rather than those specific to high-level musicality alone. Taken together, the consistency of findings reported here suggests that the dissociation of activity in medial and lateral prefrontal cortices is attributable to the experimentally constant feature of improvisation and may be a defining characteristic of spontaneous musical creativity.

## Materials and Methods

### Subjects

Six right-handed, normal hearing healthy male musicians (age range 21–50 years, mean 34.2±10.4 s.d.) participated in the study. All were full-time professional musicians (either as working performers or music professors) that were highly proficient in jazz piano playing. None of the subjects had any history of neurologic or psychiatric disorders. Informed consent was obtained for all subjects, and the research protocol was approved by the NINDS/NIDCD Institutional Review Board of the NIH.

### Improvisation Paradigms

Two block-design test paradigms were used to assess musical improvisation (see Supporting Information Audio S1, S2, S3, S4 for audio samples). The first paradigm (Scale) was designed to assess brain activity during a highly constrained paradigm of relatively low musical complexity. With a metronome playing in the background (120 beats per minute), subjects were randomly cued to play either one of two tasks. During the control task (Scale-Ctrl), subjects were instructed to play repeatedly an ascending and descending one-octave C major scale in quarter notes only, with the right hand only (Fig. 1, upper left). During the improvisation task (Scale-Improv), subjects improvised a melody in quarter notes only, but were restricted to the use of notes within the C major scale only (Fig. 1, upper right). Hence, the total number of notes, the range of those notes, the musical key, the relative technical requirements needed to play both scale and improvisation, and the acoustic content of the control and improvisation task blocks approximated one another, with the major difference being that the notes played during improvisation were spontaneously selected by the musician. Each block lasted one minute, with a total of 6 blocks (3 scale and 3 improvisation) separated by rest blocks of 30s, for a total of 9 minutes.

In the second paradigm (Jazz), a musically rich context was provided for improvisation. Prior to arrival for the scan session, all subjects received sheet music of a jazz melody (“Magnetism”, twelve-bar blues form) that was composed by one of the authors (C.J.L) to ensure novelty for the subjects (Fig. 1, lower left). The subjects memorized this melody prior to scanning, and demonstrated proficiency in playing the melody from memory prior to scanning. During scanning, a pre-recorded jazz rhythm section provided musical accompaniment. In particular, the pre-recorded music was a 12 bar blues in medium tempo (around 100 beats per minute). Two repetitions of the underlying chord progression (or “choruses”) were played in each block. During blocks, subjects were cued randomly to either play either the memorized melody (Jazz-Ctrl) or to improvise using the underlying chord progression of the novel composition (Jazz-Improv) as the basis for invention (Fig. 1, lower right). Subjects were given relative freedom during the musical improvisation blocks, with the only instruction being that the musical style of the melody and the improvisation should be consistent with one another; this instruction was intended to minimize wide variations in number of notes played, rhythmic complexity, or stylistic approach that could have been possible in an entirely unconstrained environment. Each block lasted one minute (two complete cycles of the twelve-bar chord progression), with a total of 5 control melody blocks, 5 improvisation blocks, and 9 non-performance auditory blocks, each separated by 20 s rest blocks, for a total of 25 minutes and 20 seconds. (The non-performance auditory blocks represent neural activity during listening to over-learned vs. recently generated musical passages without any active musical production or improvisation; these data are being prepared for a separate manuscript and are not discussed in the present study.)

### Piano Apparatus and Scanning Setup

A non-ferromagnetic piano keyboard (MagDesign, Redwood, CA) was custom-built with plastic keys and casing, which contained 35 full size piano keys, and sent out Musical Instrument Digital Interface (MIDI) information only (Fig. 1, upper). The MIDI information was routed to a Macintosh Powerbook G4 laptop computer using the Logic Platinum 6 musical software environment (Apple Inc., Cupertino, CA). The MIDI signal triggered a high-quality piano sample corresponding to the note played in the scanner, which was triggered using the EXS24 sampler module. The piano sound output was then routed to the subject via in-the-ear electrostatic ear speakers (Stax, Saitama, Japan), for high-fidelity reproduction of the piano sound in real-time. The piano keyboard was placed on the subjects lap in supine position, while the knees were elevated with a bolster. A mirror placed above the subjects' eyes allowed visualization of the keys during performance. Subjects were instructed to move only their right-hand during the scanning and were monitored visually to ensure that they did not move their head, trunk, or other extremities during performance. The subjects lay supine in the scanner without mechanical restraint. In addition to the electrostatic ear speakers, all subjects wore additional ear protection to minimize background scanner noise. Volume was set to a comfortable listening level that could be easily heard over the background scanner noise.

### Scanning Parameters

All studies were performed at the NMRF Imaging Facility at the NIH. Blood oxygen level dependent imaging (BOLD) data were acquired using a 3-Tesla whole-body scanner (GE Signa; General Electric Medical Systems, Milwaukee, WI) using a standard quadrature head coil and a gradient-echo EPI sequence. The scan parameters were as follows: TR = 2000 ms, TE = 30 ms, flip-angle = 90°, 64×64 matrix, field of view 220 mm, 26 parallel axial slices covering the whole brain, 6 mm thickness. Four initial dummy scans were acquired during the establishment of equilibrium and discarded in the data analysis. 270 volumes were acquired for each subject during the Scale paradigm and 760 volumes were acquired for each subject during the Jazz paradigm. In addition to the functional data, high-resolution structural images were obtained using a standard clinical T1-weighted sequence. BOLD images were preprocessed in standard fashion, with spatial realignment, normalization, and smoothing (9 mm kernel) of all data using SPM99 software (Wellcome Trust Department of Imaging Neuroscience, London, U.K.)

### Statistical Analysis

For the MIDI piano data, the total number of notes played by each subject was tabulated for each condition. The range of notes from low to high was computed for each subject by analysis of the raw MIDI data. As a quantitative measure that reflected not only the absolute range of notes but also the distribution of keyboard notes played (and to a limited extent, the physical movements required), a weighted distribution of notes was calculated. The weighted distribution was computed by taking a mean of the MIDI pitch value of all notes played (in reference to the keyboard's 35-note range), weighted by the number of times each individual note was played. Paired t-tests were used to compare piano output during control and improvised conditions for both Scale and Jazz paradigms.

For fMRI analysis, data from all six subjects were entered into a group-matrix within SPM99. Fixed-effects analyses were performed with a corrected threshold of p<0.01 (or <0.001 where noted) for significance. Contrast analyses were performed for activations and deactivations across all conditions (Improv and Ctrl), and conjunction analyses were performed for results across Jazz and Scale paradigms (p<0.01 corrected). Multi-subject conjunctions for all six subjects were also performed for each paradigm. To perform the multi-subject conjunctions, individual subject contrasts (eg. [Improvisation]–[Control]) were calculated for each subject; all individual contrasts were then subjected to a conjunction analysis without Bonferrini correction (p<0.001) that identified only those areas strictly activated (or deactivated) in all subjects [20]. For all contrasts, normalized volume coordinates from SPM were converted from Montreal Neurological Institute coordinates to Talairach coordinates for specific identification of regions of activity.

Areas of activation during improvisation were revealed by standard contrast analyses, with the application of inclusive masking of contrasts for increased specificity. Contrasts for [improvisation (I)>control (C)] were masked with contrasts for [I>rest (R)], p<0.001 corrected. This inclusive masking was used to identify areas with greater net activity during [I] than [C] attributable to increased activity during [I] within each paradigm (as opposed to decreased activity during [C]). Areas of deactivation during improvisation were revealed by inclusive masking of contrasts for [C>I] with [R>I], p<0.001 corrected; ie. areas with greater net activity during [C] than [I] attributable to deactivations during [I] within each paradigm. For example, to show activations during the Scale paradigm associated with improvisation, the contrast for [ScaleImprov>ScaleCtrl] was masked inclusively with the contrast for [ScaleImprov>ScaleRest]. An analogous method was used to identify areas of activation and deactivation associated with control conditions. Conjunction analyses were used to identify commonalities shared across paradigms for each condition. For example, to show areas activated during improvisation for both Scale and Jazz paradigms, we performed a conjunction of the results for the contrasts of [JazzImprov>JazzCtrl] masked inclusively by [JazzImprov>JazzRest] and [ScaleImprov>ScaleCtrl] masked inclusively by [ScaleImprov>ScaleRest]; the same method was applied to identify common areas of deactivation across paradigms.

## Supporting Information

Audio S1 15s excerpt of control condition, Scale paradigm
(0.26 MB WMV)

Audio S2 15s excerpt of improvisation condition, Scale paradigm
(0.26 MB WMV)

Audio S3 30s excerpt of control condition, Jazz paradigm
(0.48 MB WMV)

Audio S4 30s excerpt of improvisation condition, Jazz paradigm
(0.48 MB WMV)

Figure S1 Multi-subject conjunction analyses for Scale and Jazz paradigms. These conjunctions reveal broad deactivation of dorsolateral prefrontal cortex for both paradigms (n = 6) as well as focal activation of the medial prefrontal cortex in Jazz (n = 5) and Scale (n = 4) paradigms. Data are presented at a statistical threshold of p<0.001 without Bonferrini correction.
(7.25 MB TIF)
