# Supplementary figures and images for: Neural Substrates of Spontaneous Musical Performance: An fMRI Study of Jazz Improvisation
Source: PLoS One. 2008 Feb 27;3(2):e1679. doi: 10.1371/journal.pone.0001679 (PMC2244806; doi:10.1371/journal.pone.0001679)

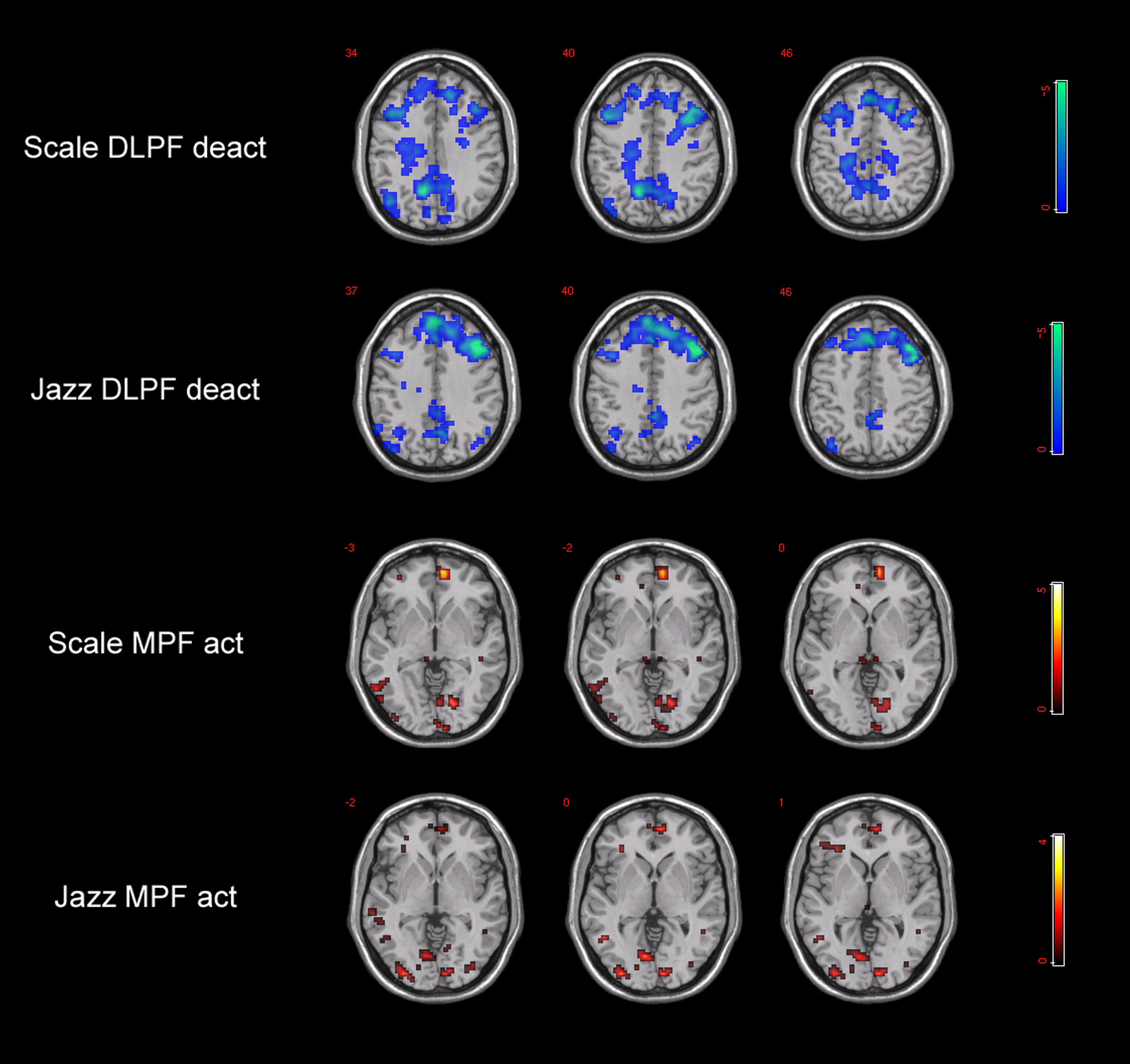

Supplement: Figure S1 — Multi-subject conjunction analyses for Scale and Jazz paradigms. These conjunctions reveal broad deactivation of dorsolateral prefrontal cortex for both paradigms (n = 6) as well as focal activation of the medial prefrontal cortex in Jazz (n = 5) and Scale (n = 4) paradigms. Data are presented at a statistical threshold of p<0.001 without Bonferrini correction. (7.25 MB TIF) [file pone.0001679.s005.tif]
